# Effects of preemptive acupuncture on cognitive function of older patients after hip replacement: a randomized controlled trial

**DOI:** 10.3389/fmed.2025.1503727

**Published:** 2025-03-13

**Authors:** Qiguo Tu, Rong Zhou, Guiping Lv, Zhengzuo Wan, Shan Chen, Bin Que

**Affiliations:** ^1^Department of Anesthesiology, Hangzhou TCM Hospital Affiliated to Zhejiang Chinese Medical University, Hangzhou, China; ^2^Operating Room, Hangzhou TCM Hospital Affiliated to Zhejiang Chinese Medical University, Hangzhou, China

**Keywords:** postoperative cognitive dysfunction, orthopedic procedures, hip replacement, inflammation, microRNA

## Abstract

**Background:**

Postoperative cognitive impairment is a common complication in older patients after major orthopedic surgery; however, the underlying mechanism is not completely understood.

**Objective:**

This study aimed to evaluate the effects of preemptive acupuncture on cognitive dysfunction after hip replacement and explore its potential mechanisms.

**Methods:**

Finally, 54 participants were randomized into sham acupuncture (*n* = 27) or acupuncture (*n* = 27) groups, who received acupuncture at the Sishencong (EX-HN1) and Baihui (DU20) acupoints, while participants in the sham acup group received sham acup at the target acupoints. Montreal Cognitive Assessment (MoCA) and Mini-Mental State Examination (MMSE) scores, the incidence of postoperative cognitive dysfunction (POCD), and other adverse events were assessed. The levels of microRNA (miR)-124 and miR-146a and inflammatory cytokines in the peripheral blood were detected. Correlations among miR-124, miR-146a, and inflammatory cytokines were analyzed.

**Results:**

Compared with the sham acup group, the MMSE and MoCA scores in the acup group on the first and seventh day after surgery were higher, and the incidence of POCD on the first day was lower. Acupuncture upregulated levels of miR-124 and -146a and decreased the levels of TNF-*α*, IL-6, and IL-1β to protect cognitive function. Correlation analysis indicated that upregulated miR-124 and miR-146 were associated with lower levels of inflammatory cytokines.

**Conclusion:**

Acupuncture protects postoperative cognitive function in older patients undergoing hip replacement, potentially reducing the incidence of POCD by upregulating miR-124 and miR-146a to inhibit neuroinflammation.

**Clinical trial registration:**

www.chictr.org.cn, identifier ChiCTR2200062027.

## 1 Introduction

With the aging world population, age-related diseases account for many medical resources. Postoperative cognitive dysfunction (POCD) is a common complication in older patients who undergo major surgeries such as cardiac and orthopedic surgeries (1). Clinically, POCD is characterized by impaired memory, decreased ability to handle information, and decreased attention, which contribute to a series of negative outcomes, including mood and personality changes. A recent review indicated that age and educational level were major risk factors for developing POCD (2). Data suggest that approximately 50% of older individuals experience at least one surgical intervention in their lifespan. In comparison, approximately 25% of older patients have an identifiable decline in cognition, and approximately 50% of those suffer from permanent cognitive impairment (3). With increasing age, older individuals inevitably suffer from neurological impairments caused by neurodegenerative diseases, such as Alzheimer’s disease.

Surgery and anesthesia are regarded as the main risk factors for POCD development. For example, various analgesics contribute to impaired cognitive function. Although short-acting opioids such as remifentanil are used for anesthesia, older patients are also vulnerable to POCD (4). However, clinical studies have indicated that even short-acting anesthetics affect postoperative cognitive impairment (5). Surgical procedures are another major risk factor for persistent postoperative pain and systemic inflammation raised during surgery. For example, after orthopedic surgery, patients often experience persistent pain that contributes to microglia activation, promotes cytokine release, and impairs the normal function of neurons (6, 7). However, the specific physiological mechanism of postoperative cognitive impairment remains unclear, and optimal perioperative strategies and treatments are required to prevent POCD. There are multiple pharmacological applications in clinical practice for the postoperative prevention of cognitive dysfunction, including glucocorticoids. A recent 4-year follow-up study suggested that the administration of 0.1 mg·kg^−1^ dexamethasone effectively reduced the incidence of POCD after cardiac surgery (8). Pharmacological treatments may also lead to other side effects and increase hospital costs. However, non-pharmacological applications are lacking.

Acupuncture is a traditional Chinese treatment or technique for anesthesia, lower back pain control, and prevention of chemotherapy-related side effects (9). Patients often experience preoperative anxiety, which is not conducive to rapid postoperative recovery. A recent meta-analysis suggested that acupuncture contributed to reducing preoperative anxiety and pain intensity (3), demonstrating the positive effects of acupuncture in patients during the preoperative period. However, further studies are needed to verify this. Surgical trauma and anesthesia-mediated neuroinflammation may be the underlying mechanisms of postoperative cognitive dysfunction. Animal experiments have shown that electroacupuncture on ST25 and ST36 acupoints inhibited the release of TNF-*α* induced by endotoxemia, improved the survival rate of septic rats (10), and elucidated the anti-inflammatory effects of acupuncture on the central circuit. The optimal effects of acupuncture were determined by optimal acupoint choice. According to the theory of Traditional Chinese Medicine (TCM), acupuncture in DU20 is widely used to treat dementia, stroke, insomnia, epilepsy, and hypertension. One study suggested that electroacupuncture at DU20 helped improve cognition (11). The above evidence supports the clinical use of DU20 in cognitive protection.

Acupuncture with EX-HN1 contributes to lower glial fibrillary acidic protein (GFAP)-positive astrocytes in the hippocampus, inhibits oxidative stress, and improves cognitive function in perioperative neurocognitive disorders (PNDs) in rats (4). Recently, non-coding small RNA molecules, including miRNAs, have been shown to play an important role in the pathophysiology of various neurodegenerative diseases. Some have been shown to regulate neuronal function and neuroinflammation and serve as biomarkers of neuroinflammation (6). A recent study has indicated that miR-124 is a novel target for regulating microglial activation, thus mediating postoperative cognitive function (12). Upregulation miR-146a expression inhibits the release of proinflammatory cytokines (TNF-*α*, IL-1β, and IL-6), thus attenuating hippocampus-dependent learning and memory impairment in mice with POCD (13). However, it remains unclear whether acupuncture alleviates postoperative cognitive impairment by regulating these miRNAs. This study aimed to investigate the effects of acupuncture on postoperative cognitive function and neuroinflammation and to elucidate the underlying mechanisms.

In this study, we hypothesized that the preemptive application of acupuncture might effectively inhibit the inflammatory response by upregulating miRNA-124 and -146a, thus reducing neuroinflammation and improving cognitive function in older individuals after hip replacement.

## 2 Materials and methods

### 2.1 Participant enrollment

This randomized controlled trial was conducted following the Helsinki Declaration and Good Clinical Practice Guidelines and was approved by the Medical Ethics Committee of Hangzhou TCM Hospital, Zhejiang Chinese Medical University (No. 2022KY045). The protocol used in this study was registered with the China Clinical Trial Registry (No. ChiCTR2200062027). Sixty-six participants underwent hip replacement between 20 July 2022 and 20 December 2022. All participants provided informed consent prior to the surgery. The study participants were scheduled for hip replacement under combined spinal-epidural anesthesia (CSEA). The eligibility criteria of the study are as follows: (1) aged ≥65 years old, (2) American Association of Anesthesiologists (ASA) status ≤ grade III, and (3) agreed to receive acupuncture. Exclusion criteria are as follows: (1) without full communication skills, (2) without the ability to finish the Montreal Cognitive Assessment (MoCA) and Mini-Mental State Examination (MMSE) questionnaires or preoperative MoCA scored <26 points or MMSE <24 points, (3) with history of uncontrolled hypertension, diabetes, or other coronary system diseases, (4) target acupuncture sites were not applicable for acupuncture, (5) participants not suitable for CSEA, (6) with history of acupuncture treatment or related acupoint stimulation treatments (such as acupoint electrical stimulation, and nerve electrical stimulation), and (7) participants cannot tolerate *de qi* sensation.

### 2.2 Randomization and blinding protocol

We first numbered the participants according to the order of hospitalization, matched the above number with a random number, and divided the participants into sham acup group and acup group based on the above random numbers. Randomization was performed by an independent statistician blinded to the other trial researchers. Before surgery, an independent blinded statistician concealed the file that provided the participants with grouping information. The collected and assessed outcomes were blinded to the group information. The participants were treated separately during hospitalization to avoid mutual communication. All the participants were allowed to drop out at any time during the study period.

### 2.3 Intervention of acupuncture and anesthesia protocol

**Figure 1:**
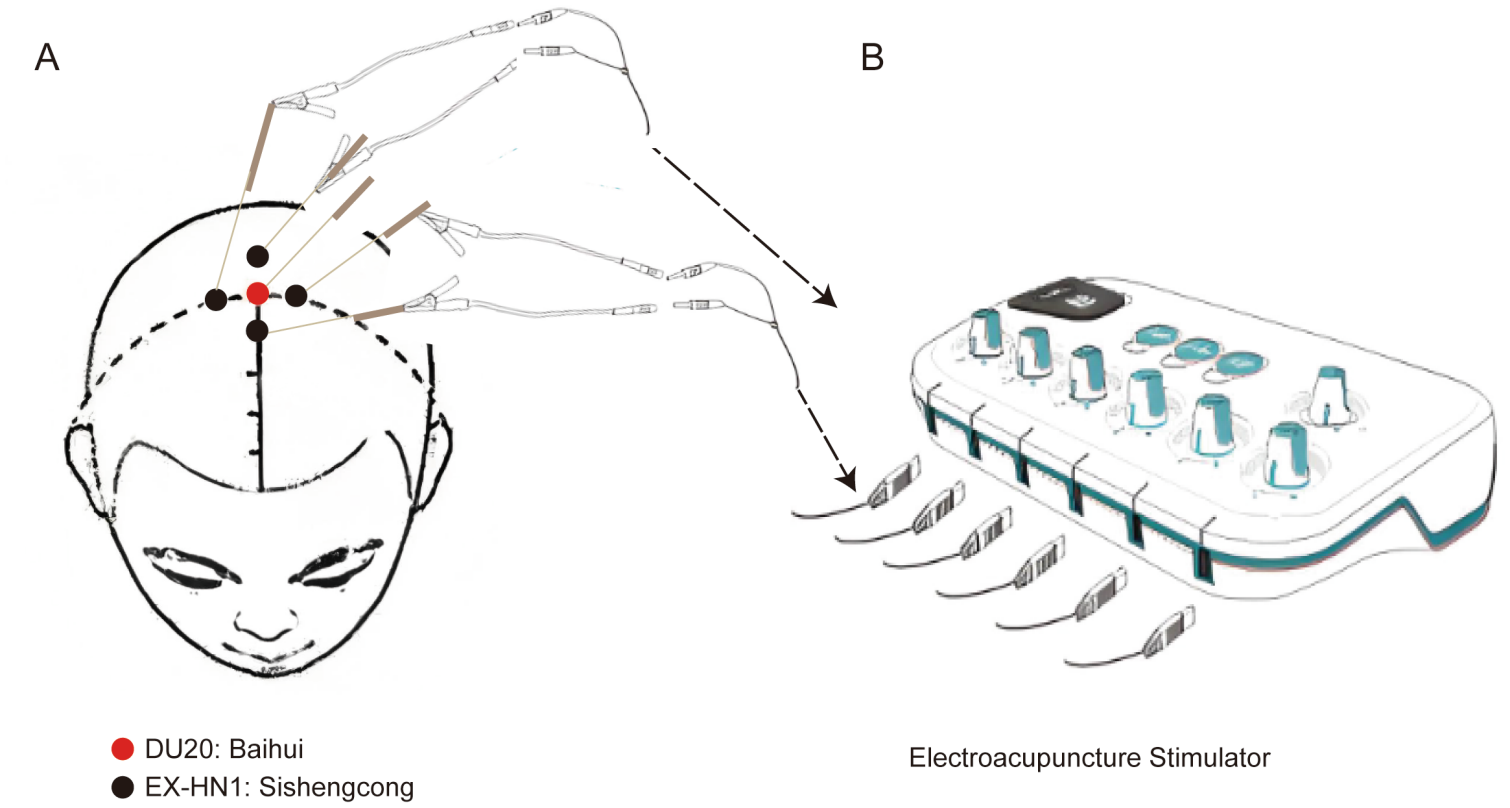
Acupoint diagram of target acupoints. **(A)** Anatomical location of DU20 and EX-HN1. **(B)** Electroacupuncture stimulator connects the electric needles on the target acupoints through a wire. DU20, locating at the intersection between the midline of the head and the connecting line between the two ear tips. EX-HN1, composing of four acupoints, away 3.33 cm on the front, back, left and right of DU20.

Before the study, the participants were well informed about the acupuncture procedure and associated adverse events. Acupuncture was performed on DU20 (located at the intersection between the midline of the head and the connecting line between the two ear tips) and EX-HN1 (composed of four acupoints, away 3.33 cm on the front, back, left, and right of DU20) according to participant grouping information in an envelope (Figure 1), which was only available to the acupuncture operator, who was not permitted to participate in data collection and analysis. Based on our experience and our previous study (14), acupuncture needles (0.25 mm, Dongbei Medical Equipment Co., Ltd., Jinan, China) were inserted at the target acupoints, with stimulation for 30 min (a distant-dense wave with a frequency of 2/10 Hz), three times a day (07:00, 13:00, and 19:00), and 3 consecutive days before surgery. *De qi* sensation is a landmark for the successful implementation of acupuncture (15). Acupuncture was considered successful when a *de qi* sensation was obtained. The needle was maintained for 30 min, connected to an acupoint stimulator (dense-disperse frequency, 2/10 Hz, 6–9 mA), and carefully protected from removal. For participants in the sham acup group, needles were inserted into the skin approximately 1 cm away from the target acupoints and connected to the acupoint stimulator; however, stimulation was not performed. Once *a de qi* sensation was obtained, participants were excluded from the group. The operator also protected the needles.

A standard CSEA protocol was used to provide anesthesia for the hip replacement. A routine intravenous cannula was inserted, and 10 mL/kg of lactated Ringer’s solution was administered. Vital signs, including invasive blood pressure (BP), heart rate (HR), respiratory rate (RR), electrocardiographic monitoring (ECG), and blood oxygen saturation (SpO2), were intensively monitored throughout surgery. Before CSEA, the patient was placed on the right arm and reclined with the hands holding the knees. After skin sterilization, 5 mL of 2% lidocaine was used for local infiltration anesthesia. An 18G needle was advanced between the mid-line of the L2–3 or L3–4 intervertebral space, and a loss-of-resistance technique with saline was used to test the reattachment of the epidural space. A 27-G pencil-point needle was inserted using the needle-through-needle technique. Two volumes of 2 mL of 0.5% bupivacaine were administered in a single dose over half a minute. The epidural catheter was advanced approximately 3 cm into the epidural space, the spinal needle was removed, negative pressure was checked, and the catheter was carefully protected throughout the surgery. A single dose of 2 mL of 0.375% ropivacaine was administered to maintain anesthesia. The patients were placed in the supine position after CSEA. At the end of the surgery, 2 mg of morphine was administered for postoperative analgesia.

### 2.4 Measurements

POCD was diagnosed according to a previous study based on the MoCA and MMSE scale assessment (16, 17). The incidence of POCD within 7 days after surgery was documented. Peripheral levels of miR-124 and miR-146a, as well as inflammatory cytokines in the periphery, were detected.

The MMSE and MoCA scores were recorded 1 day before surgery and on the first and seventh days after surgery by a nurse anesthetist, blinded to the grouping information. When postoperative MMSE and MoCA scores were reduced by more than one standard deviation compared to baseline values, POCD was diagnosed (7, 18). A volume of 2 mL of peripheral blood was collected after surgery for detecting the levels of miR-124 and miR-146a by Quantitative real-time PCR and levels of inflammatory cytokines of TNF-*α*, IL-6, and IL-1β by ELISA method (R&D system, California, USA) according to the manufacturer’s instructions.

### 2.5 Statistical analysis

The sample size was calculated using a web tool^1^ based on our preliminary study on the incidence of POCD. In brief, a sample size of 20 participants for each group was obtained to reach a significance difference of *α* = 5%, with a statistical power (*β*-value) of 0.8. We also assumed a 20% dropout rate; therefore, the total sample size of the study was 48.

Data were shown as mean ± standard deviation (SD) for continuous variables or numbers and proportions for categorical variables. The ANCOVA was used to compare the differences between the two groups, and rmANOVA was used for repeated measures. Mixed linear models analyzed the correlation between miR-124, miR-146a, and inflammatory cytokines. Chi-square (and Fisher’s exact) tests were used to analyze categorical variables. A *p*-value of <0.05 was considered statistically significant. SPSS software (version 18.0) was used to analyze the data.

## 3 Results

### 3.1 Demographic characteristics of participants

**Figure 2:**
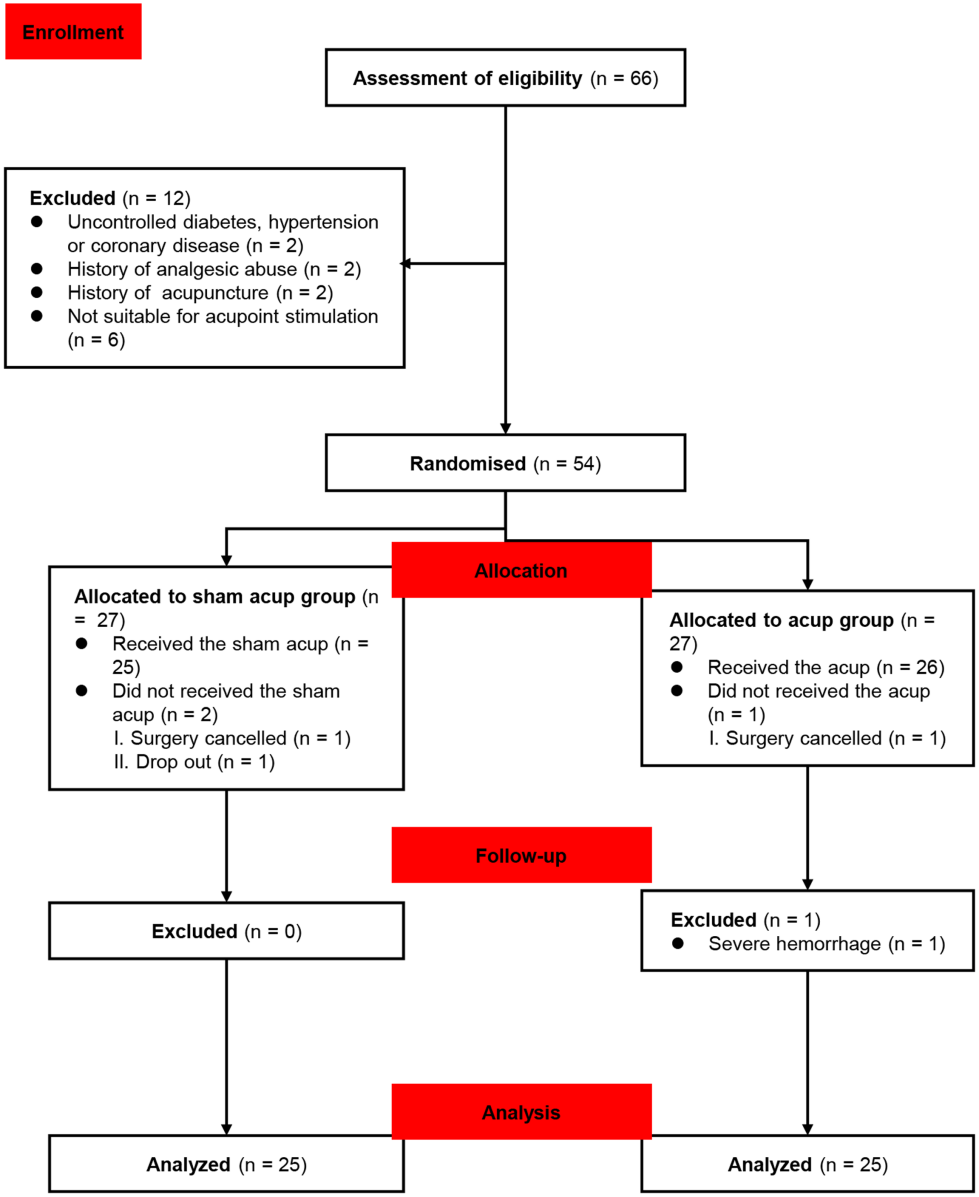
Flow chart of participants enrollment and randomization.

Sixty-six participants were enrolled in the study from 20 July 2022 to 20 December 2022. Twelve patients were excluded as they were ineligible for the study. One of them had uncontrolled hypertension and diabetes (the participant had not been diagnosed); 1 of them had coronary disease, diagnosed through auxiliary examinations after admission; 2 of them had a history of abuse of analgesics, which may potentially affect cognitive function during the perioperative period; 2 of them had a history of other types of acupuncture treatment; 6 of them were found unsuitable for acupuncture, and 54 participants were randomized into the sham acup and acup groups. After allocation, two participants did not receive sham acup for cancellation of the surgery, as well as one surgical cancellation was performed in the acup group. During follow-up, one participant dropped out because of a severe hemorrhage during surgery. Fifty participants completed the study and were included in the data analysis (Figure 2). The baseline characteristics of the groups are shown in Table 1.

**Table 1:** Baseline characteristics of participants.

Characteristics	Sham acup (*n* = 25)	Acup (*n* = 25)	*p*-value
Demographic variables			
Age [year; mean ± SD]	68.56 ± 2.72	70.04 ± 4.56	0.17
Gender [*n* (%)]			0.779
Female	13 (52)	12 (48)	
Male	12 (48)	13 (52)	
BMI [kg/m^2^, mean ± SD]	23.2 ± 1.96	23.24 ± 2.55	0.9503
ASA status [*n* (%)]			0.5688
II	12 (48)	10 (40)	
III	13 (52)	15 (60)	
Education [years, mean ± SD]	9.32 ± 3.89	7.64 ± 3.4	0.1107
Alcohol intake [years, mean ± SD]	7.08 ± 7.51	7.32 ± 8.45	0.9159
Duration of surgery [min, mean ± SD]	121.08 ± 14.5	121.32 ± 13.72	0.9523
Duration of anesthesia [min, mean ± SD]	140.4 ± 12.79	146.24 ± 12.87	0.1140
Blood loss [mL, mean ± SD]	119.76 ± 35.09	126.7 ± 50.09	0.5746
Alcohol intake [years, mean ± SD]	7.08 ± 7.51	7.32 ± 8.45	0.9159

Values are presented as mean ± SD or n (%). SD, standard deviation, BMI, body mass index; ASA, American Society of Anesthesiologists.

### 3.2 MMSE, MoCA assessment, the incidence of POCD, and adverse events

Except for the MoCA score on the seventh day, the MMSE and MoCA scores in both groups declined compared to those recorded before the surgical procedure. The MMSE scores on the first (*p* = 0.0002) and seventh (*p* = 0.0027) were higher in the acup group than in the sham acup group. In addition, the MoCA scores in the acup group were higher than those in the sham acup group on the first (*p* = 0.0006) and seventh (*p* = 0.0342) days after surgery. The incidence of POCD in the acup group was lower on the first day (*p* = 0.0322), but not on the seventh (*p* = 0.1706) day after surgery, compared with the sham acup group. We also documented the adverse effects of acupuncture at the target site. Pain, pruritus, and redness were common side effects of acupuncture; however, no significant differences were observed between the groups (Table 2).

**Table 2:** MMSE and MoCA scores and incidence of POCD, adverse events associated with acupuncture.

Variables	Sham acup (*n* = 25)	Acup (*n* = 25)	*p*-value
MMSE score			
1 day pre-surgery	27.04 ± 0.79	27.12 ± 0.83	0.689
First post-surgery	23.4 ± 1.16	25.52 ± 1.26	0.0002
Seventh post-surgery	25.48 ± 0.92	26.36 ± 0.99	0.0027
MoCA score			
1 day pre-surgery	27.56 ± 1.25	26.43 ± 1.73	0.3367
First post-surgery	23.64 ± 1.37	25.87 ± 1.19	0.0006
Seventh post-surgery	25.33 ± 0.25	26.79 ± 0.26	0.0342
POCD (*n*, %)			
First post-surgery	12 (48%)	4 (16%)	0.0322
Seventh post-surgery	8 (32%)	3 (12%)	0.1706
Adverse events of acupuncture (n, %)			
Pain	3 (12%)	1 (4%)	0.6092
Pruritus	2 (8%)	4 (16%)	0.6671
Skin redness	1 (4%)	4 (4%)	0.3484

Values are presented as mean ± SD, or n (%). MoCA, Montreal Cognitive Assessment; POCD, Postoperative cognitive dysfunction.

### 3.3 Levels of microRNAs in peripheral blood

**Figure 3:**
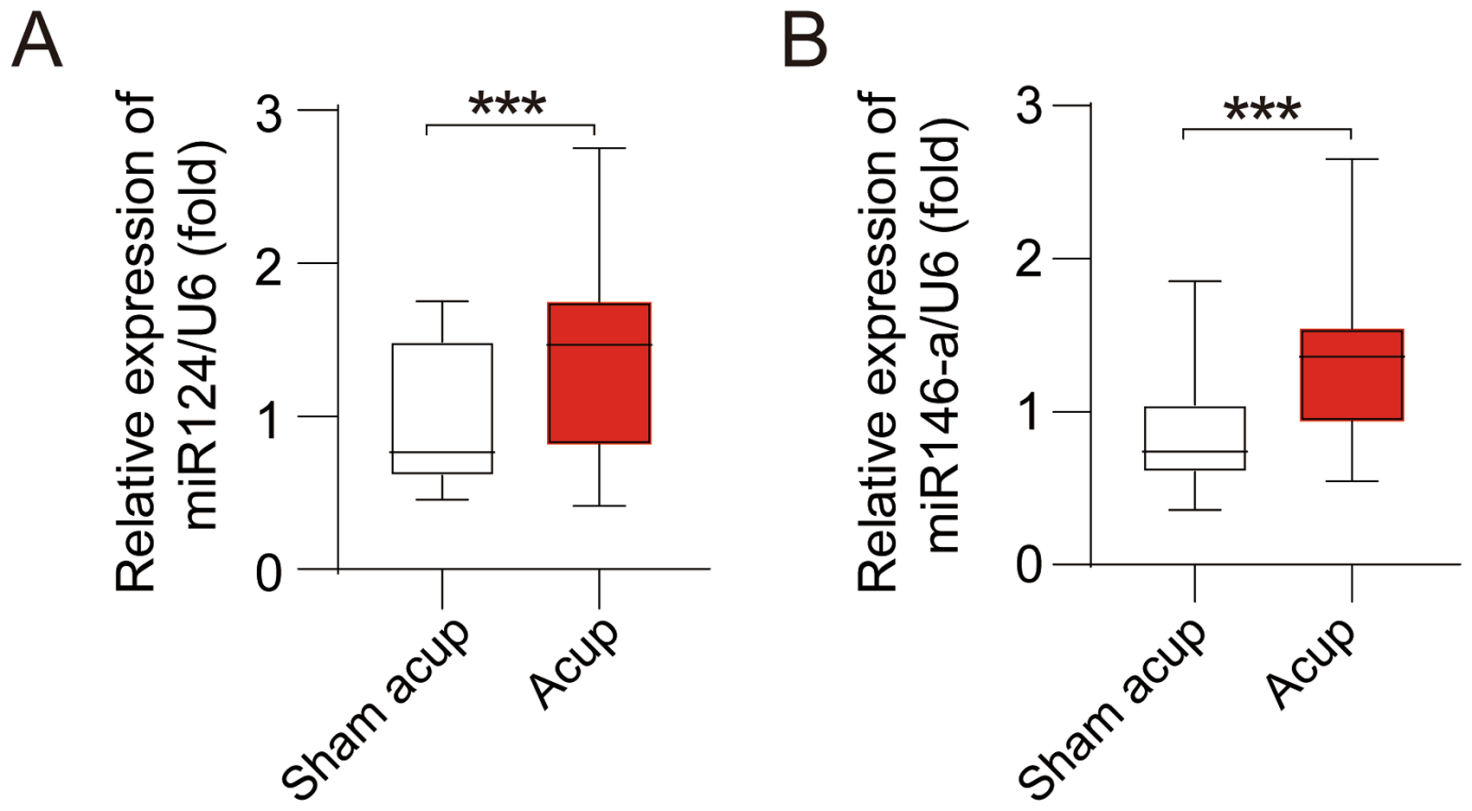
Levels of miRNAs in in peripheral of participants treated with sham acupuncture or acupuncture. **(A)** Levels of miR124 in peripheral; **(B)** Levels of miR146-a in peripheral. ***p < 0.001.

MiR-124 and miR-146a have been suggested to protect cognitive function and anti-neuroinflammation (12, 13). We detected peripheral blood levels of miR-124 and miR-146a. Compared with patients treated with sham acup, those treated with acupuncture showed higher levels of miR-124 (Figure 3A) and miR-146a (Figure 3B).

### 3.4 Levels of inflammatory cytokines in peripheral blood

**Figure 4:**
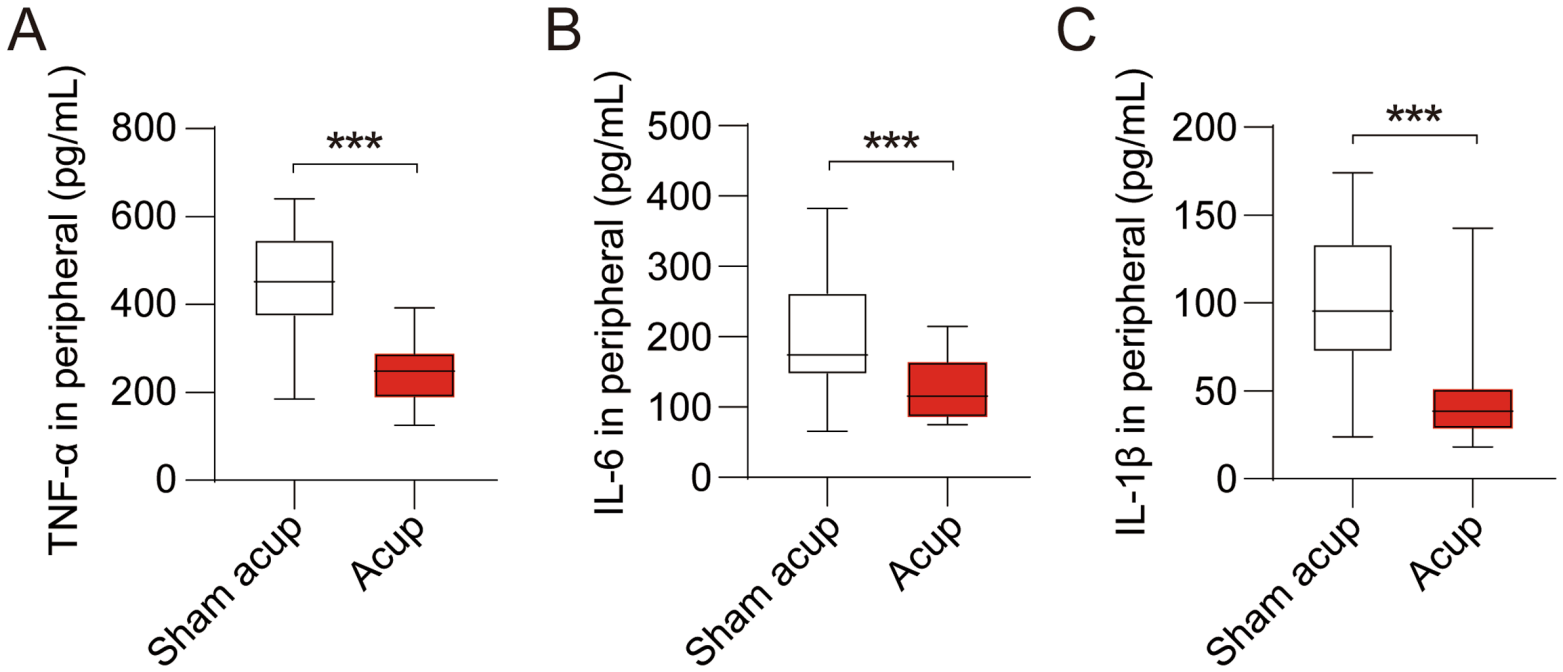
Levels of inflammatory cytokines in peripheral of participants treated with sham acup or acup. **(A)** Levels of TNF-α in peripheral; **(B)** Levels of IL-6 in peripheral; **(C)** Levels of IL-1β in peripheral. ***p < 0.001.

Neuroinflammation is a major cause of perioperative cognitive impairment. Compared with the sham acup group, patients treated with acupuncture showed lower levels of TNF-*α* (Figure 4A), IL-6 (Figure 4B), and IL-1β (Figure 4C), suggesting that acupuncture inhibited the inflammatory response during the perioperative period, which might help reduce the neuroinflammation and thus to alleviate cognitive impairment.

### 3.5 Upregulation of miR-124 and miR-146a associated with the low level of inflammatory response

**Figure 5:**
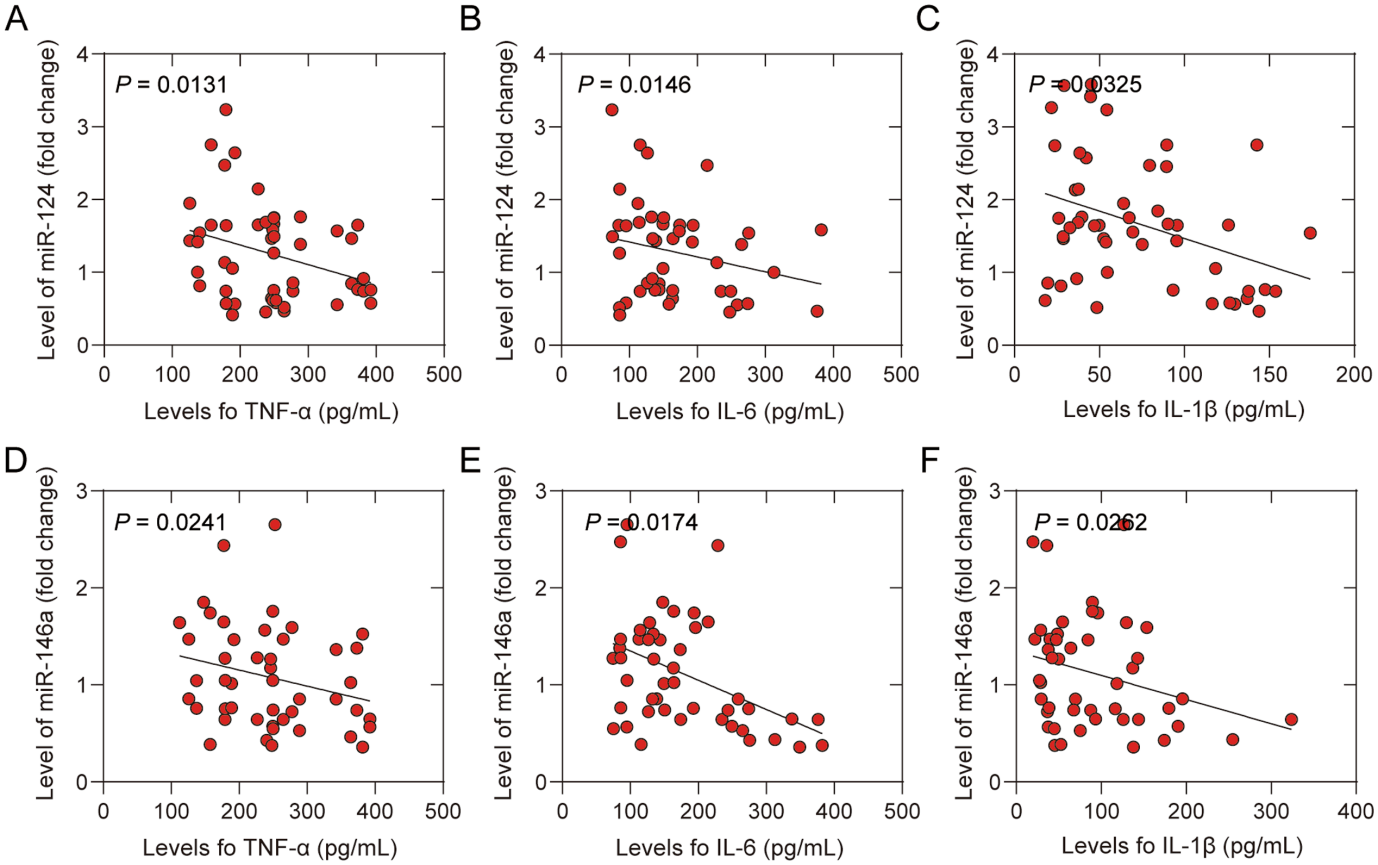
Correlation between levels of inflammatory cytokines in peripheral and microRNAs of participants treated with sham acup or acup. **(A)** Correlation between levels of TNF-α and miR-124 in peripheral; **(B)** Correlation between levels of IL-6 and miR-124 in peripheral; **(C)** Correlation between levels of IL-1β and miR-124 in peripheral; **(D)** Correlation between levels of TNF-α and miR-146a in peripheral; **(E)** Correlation between levels of IL-6 and miR-146a in peripheral in peripheral; **(F)** Correlation between levels of IL-1β and miR-146a in peripheral.

These results indicated that acupuncture upregulated the levels of miR-124 and miR-146a and inhibited the inflammatory response in older patients after hip replacement. Animal experiments have shown that miRNAs regulate inflammatory responses in some disease models (12, 13). To reveal the potential mechanisms, we conducted a correlation analysis between the levels of miR-124 and miR-146a and the levels of inflammatory cytokines. The results suggested that upregulated miR-124 (Figures 5A–C) and miR-146a (Figures 5D–F) are associated with lower levels of inflammatory cytokines in peripheral blood, including TNF-*α*, IL-6, and IL-1β. These results suggested that acupuncture inhibited the inflammatory response by upregulating the expression of miR-124 and miR-146a.

## 4 Discussion

The results of the present study suggested that acupuncture decreased the incidence of POCD in the early postoperative period after hip replacement. Mechanistically, acupuncture alleviated the postoperative inflammatory response by upregulating miR-124 and miR-146a levels. Correlation analysis indicated that the upregulation of miR-124 and miR-146a, associated with a decreased release of inflammatory cytokines, might help reduce neuroinflammation, thus alleviating cognitive impairment. These results suggest potential mechanisms by which acupuncture inhibits the inflammatory response, contributing to cognitive protection during the perioperative period.

However, pathophysiological mechanisms underlying POCD remain unclear. The risk factors include age, educational level, and mental health. For patients undergoing surgical procedures, anesthesia and surgery may further deteriorate postoperative cognitive function. A recent study suggested that approximately 50% of older patients experienced cognitive decline on the first day after a total hip arthroplasty (19). Early prevention and management of POCD are crucial for its prognosis. However, the medical treatment is often delayed. Therefore, the application of preventive measures before surgery may be beneficial. Recent evidence has shown that acupuncture contributes to the alleviation of POCD by inhibiting inflammatory responses and oxidative stress (20). In this study, we selected DU20 and EX-HN1 as the target acupoints.

DU20 is widely used to treat dementia, stroke, insomnia, epilepsy, and hypertension. Studies have suggested that electroacupuncture at DU20 could help improve cognitive function (10, 11). These findings offer guidance for the clinical application of DU20 in enhancing cognitive function. Acupuncture with EX-HN1 has been shown to have calming effects and to improve cognitive abilities (21). Another study indicated that acupuncture of EX-HN1 contributes to reducing the GFAP GFAP-positive astrocytes in the hippocampus, mitigating oxidative stress, and enhancing the cognitive function of PNDs in rats (5). A recent meta-analysis suggested that acupuncture could successfully treat and prevent POCD (22). However, the underlying mechanism remains unclear. In early 1975, the MMSE emerged as the preferred tool for the clinical evaluation of cognitive function in older individuals (23). It is simple and convenient to operate, leading to its widespread use worldwide, and has become the main scale to evaluate postoperative cognitive impairment (17). The MoCA questionnaire is frequently used to evaluate postoperative cognitive function (16). In the current study, the MMSE and MoCA scores declined after surgery. However, patients who received acupuncture experienced higher MMSE and MoCA scores and a lower incidence of POCD in the early postoperative period, consistent with the findings of a previous study (18). These results suggest that perioperative acupuncture at DU20 and EX-HN1 may enhance cognitive function in older patients undergoing hip replacement.

Neuroinflammation is a significant factor in the development of POCD (24). Research involving animal models has indicated that surgical procedures increased pro-inflammation in the peripheral and central nervous systems, ultimately resulting in neuronal injury (25, 26). Another study revealed that lipopolysaccharide administration before surgery increases the incidence and severity of POCD (27). This evidence suggests that peripheral and central inflammation contribute significantly to cognitive dysfunction. A previous study demonstrated that acupuncture could effectively inhibit oxidative stress and inflammation (28), potentially mitigating neuroinflammation during surgical procedures. However, existing evidence remains insufficient. In this study, anesthesia and surgery were associated with a pronounced release of inflammatory cytokines, as evidenced by increased TNF-*α*, IL-6, and IL-1β levels. Conversely, patients who received acupuncture exhibited lower levels of inflammatory responses, indicating that acupuncture may exert anti-inflammatory effects that could help reduce cognitive impairment and the incidence of POCD.

A recent review indicated a strong association between miRNAs, neuroinflammation, and cognitive impairment (29). A few miRNAs have been identified to be influential in the development of POCD (30). Furthermore, miRNAs have recently been recognized as significant bridges related to the effects of acupuncture, although their biological mechanisms remained unclear (31). MiR-124 is one of the most abundant miRNAs in the brain and regulates the function of microglia (12). MiR-146a is upregulated in neurodegenerative diseases and is involved in modulating the function of microglia (32). This indicates that miR-124 and miR-146a may influence cognitive function and possess anti-neuroinflammatory properties (12, 13). Therefore, in the present study, we examined the levels of miR-124 and miR-146a. Acupuncture triggered the expression of both miR-124 and miR-146a, and patients who received acupuncture exhibited lower inflammatory levels. Correlation analysis showed that the upregulation of miR-124 and miR-146a was associated with decreased levels of inflammatory cytokines, implying that acupuncture may mitigate the inflammatory response by upregulating miR-124 and miR-146a.

Despite favorable outcomes, the limitations of this study must be acknowledged. First, the participant cohort was drawn from a single center, indicating the need for a more diverse sample encompassing various races and nationalities to enhance the generalizability of the findings. Second, other factors, such as postoperative pain, may contribute to the triggering of inflammatory responses. Various studies have reported that acupuncture is widely used as an analgesia (27, 28). Third, POCD mainly occurs within 7 days after anesthesia and surgery (33), and we only recorded the MMSE and MoCA scores within 7 days after surgery. Given that POCD may affect the long-term prognosis of patients, including cognitive function and quality of life, further research is required to evaluate the long-term effects of acupuncture on postoperative cognitive impairment. Fourth, the implementation of acupuncture in this study may not have been optimal. We intend to refine this operational process further to decrease the associated labor costs. Correlation analysis indicated that the upregulated miR-124 and miR-146a levels were associated with reduced levels of inflammatory cytokines. However, further investigations are required to confirm this, especially through *in vitro* experiments. We designed an animal experiment to test this hypothesis. In this study, the acupuncturists could not be blinded to the nature of the intervention. This may have introduced a performance bias. However, the acupuncturists were not allowed to participate in the data collection and analysis. Pain, pruritus, and redness of the target acupoints were the most common complications. Therefore, we mainly included the incidence of these three complications. More potential complications or side reactions would be beneficial for a comprehensive safety assessment. During registration, we only listed the main outcomes, such as MMSE scores, levels of miR-124, and inflammatory cytokines. However, a detailed assessment of outcomes should be included in the trial registration. Using multiple methods to assess the postoperative cognitive function makes these results more convincing. Finally, the study is a single-center study, which may affect the generalizability of the findings. Therefore, more strictly designed studies, including more centers and results involving long-term effects, are needed to confirm the results of the study further.

## 5 Conclusion

This study indicated that the proactive application of acupuncture at the DU20 and EX-HN1 points in older patients undergoing hip replacement surgery effectively enhanced postoperative cognitive function, improved MMSE and MoCA scores, and decreased the incidence of POCD in the early postoperative period. Mechanistically, acupuncture upregulates the levels of miR-124 and miR-146a, which play roles in mitigating neuroinflammation, thereby facilitating improvements in postoperative cognitive function. However, owing to certain limitations, further rigorously designed studies are necessary to validate the findings of this study.

## Data Availability

The original contributions presented in the study are included in the article/supplementary material, further inquiries can be directed to the corresponding author.

## Footnotes

^1^ https://sample-size.net
